# Unilateral Proptosis and Bilateral Compressive Optic Neuropathy in a Meningioma Patient

**DOI:** 10.7759/cureus.53728

**Published:** 2024-02-06

**Authors:** Tan Teng Siew, Shahidatul-Adha Mohamad, Rafidah Sudarno, Vithiaa Nilamani

**Affiliations:** 1 Department of Ophthalmology and Visual Science, Universiti Sains Malaysia, School of Medical Sciences, Kota Bharu, MYS; 2 Department of Ophthalmology, Hospital Tengku Ampuan Rahimah, Klang, MYS; 3 Department of Ophthalmology, Hospital Universiti Sains Malaysia, Kota Bharu, MYS; 4 Department of Ophthalmology, Hospital Kuala Lumpur, Kuala Lumpur, MYS

**Keywords:** meningioma, compressive optic neuropathy, optic neuropathy, unilateral proptosis, proptosis

## Abstract

Unilateral proptosis can be a sign of a potential threat to vision or life. Here, we report a case of unilateral proptosis with bilateral asymmetrical compressive optic neuropathy. A 36-year-old Malaysian indigenous female presented with painless right-eye proptosis associated with progressive blurring of vision for the past month. She had painless progressive left-eye vision loss for eight years. There was marked right-eye proptosis with partial ophthalmoplegia. The optic nerve functions were significantly reduced in the left eye with a positive relative afferent pupillary defect (RAPD). Humphry perimetry showed a right superior nasal field defect. Brain imaging showed two different masses located at the suprasellar and right greater wing of the sphenoid extraaxial lesion likely representing a meningioma. She was diagnosed with bilateral compressive optic neuropathy secondary to intracranial mass and was referred to the neurosurgical team for further intervention. This case highlights that painless proptosis with early vision loss of the fellow eye may be the early presenting symptom of meningioma, without any symptoms of raised intracranial pressure. Brain imaging is warranted to rule out any intracranial pathology if a visual field defect is present.

## Introduction

Unilateral proptosis is a term indicating the protrusion of a single eye and can arise from various pathologies, which may pose risks to vision or life. Clinically, unilateral proptosis may present with pain or be painless, and its progression can vary in speed depending on its underlying pathology. Differential diagnosis of unilateral proptosis is extensive, encompassing infectious, inflammatory, vascular, and neoplastic conditions, such as intra-orbital or intracranial mass like meningioma [1].

Meningiomas are a common benign tumor that develop in the central nervous system (CNS). They constitute 53.2% of non-malignant primary CNS tumors [2]. Women are more significantly impacted compared to men with a ratio of 3:2 [2]. Individuals with a strong family history of meningioma and neurofibromatosis-2 (NF-2) gene mutation are at an increased risk for meningioma [3]. Ophthalmic manifestations are observed in up to 50% of patients diagnosed with a primary brain tumor [4,5]. In this article, we describe a case of unilateral proptosis with bilateral asymmetrical compressive optic neuropathy secondary to meningioma, which presented with early visual loss in one eye with a contralateral proptosis eye. We obtained informed consent from the patient to document this case report.

## Case presentation

A 36-year-old Malaysian indigenous female presented with painless right-eye proptosis associated with progressive blurring of vision for the past month. She had a history of painless progressive left-eye vision loss for eight years. There were no signs of eye redness or diplopia. She denied any symptoms of hyperthyroidism, no high intracranial pressure symptoms, no constitutional symptoms, and no family history of malignancy.

**Figure 1:**
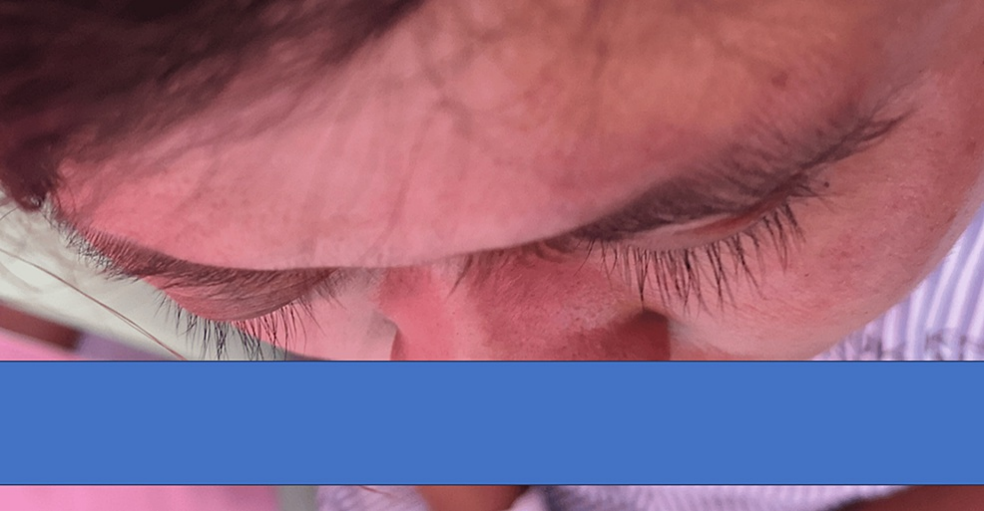
Obvious protrusion of the right eye.

Her best-corrected vision was 6/9 on the right and hand motion (HM) on the left. There was marked right eye proptosis (Figure 1).

**Figure 2:**
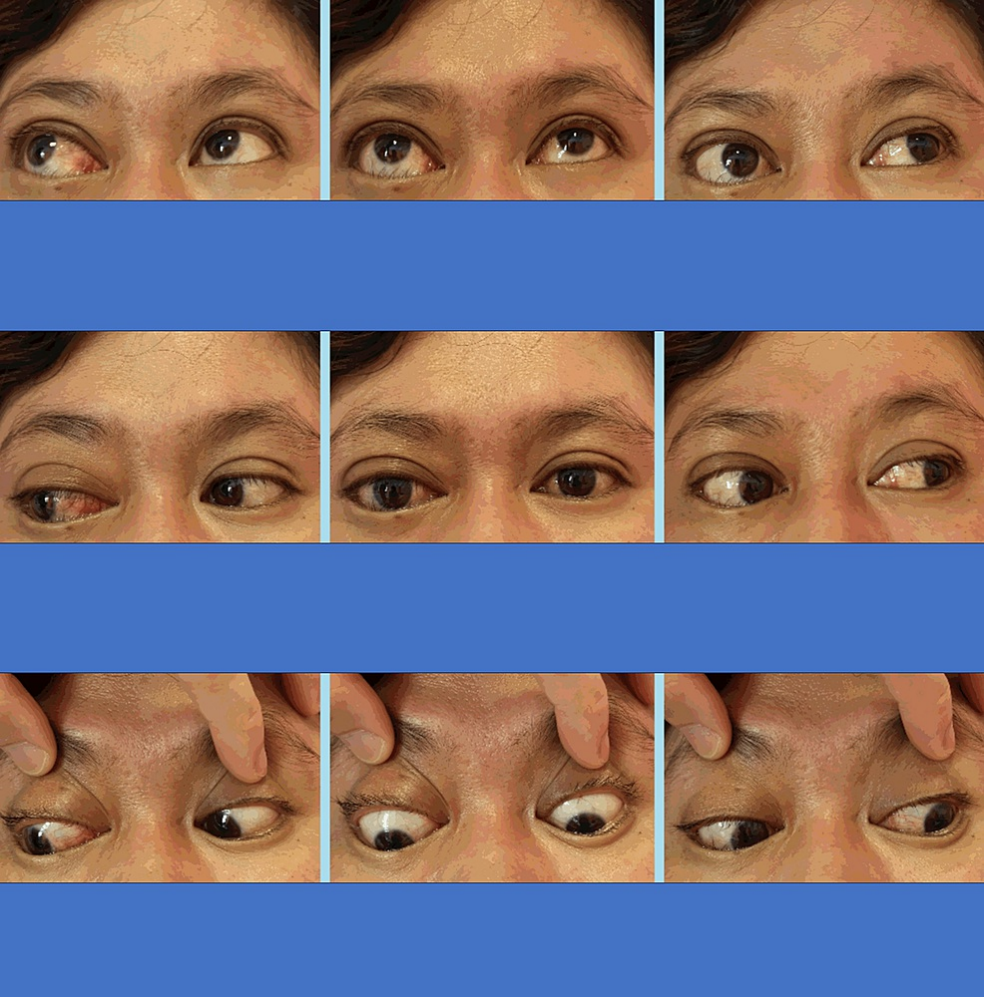
Nine cardinal gazes demonstrate a slight limitation of right-eye movement on the left gaze.

Hertel’s exophthalmometry showed a difference of 8 mm between the right and left eyes with right partial ophthalmoplegia over adduction and superonasal gaze (Figure 2).

**Figure 3:**
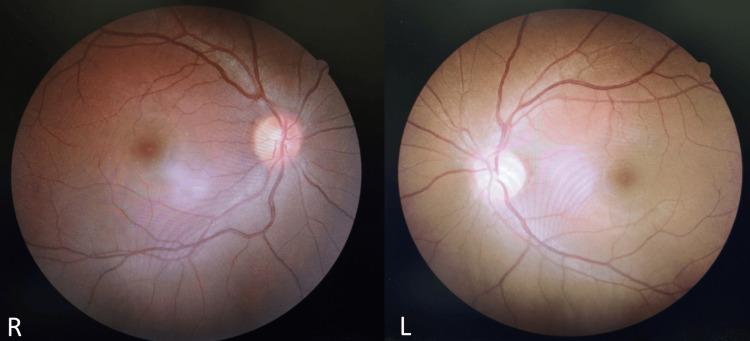
Right eye's normal fundus photo (R). Left eye's optic disc color was pallor, indicating the presence of optic atrophy (L).

The optic nerve functions were significantly reduced in the left eye with a positive relative afferent pupillary defect (RAPD). Both eyes’ intraocular pressure was normal. Examination of the anterior was otherwise unremarkable for both eyes. The right eye’s optic disc was pink with a cup-to-disc ratio (CDR) of 0.3 but no signs of optic disc swelling. The left eye’s optic disc was mild pallor in color with CDR 0.3 indicating signs of optic atrophy (Figure 3).

Humphrey perimetry showed a right superonasal field defect, and the left eye was unable to perform a visual field test due to severe vision loss secondary to chronic optic atrophy. Systemic examination was unremarkable.

Baseline blood tests, including full blood count and liver and renal function tests, were normal. Serum tests for hormones, such as follicle-stimulating hormone (FSH), luteinizing hormone (LH), cortisol, prolactin, and thyroid function, yielded normal results. Viral screenings including human immunodeficiency virus (HIV), hepatitis B and C, and venereal disease showed non-reactivity. Her serum tumor markers were normal as well.

**Figure 4:**
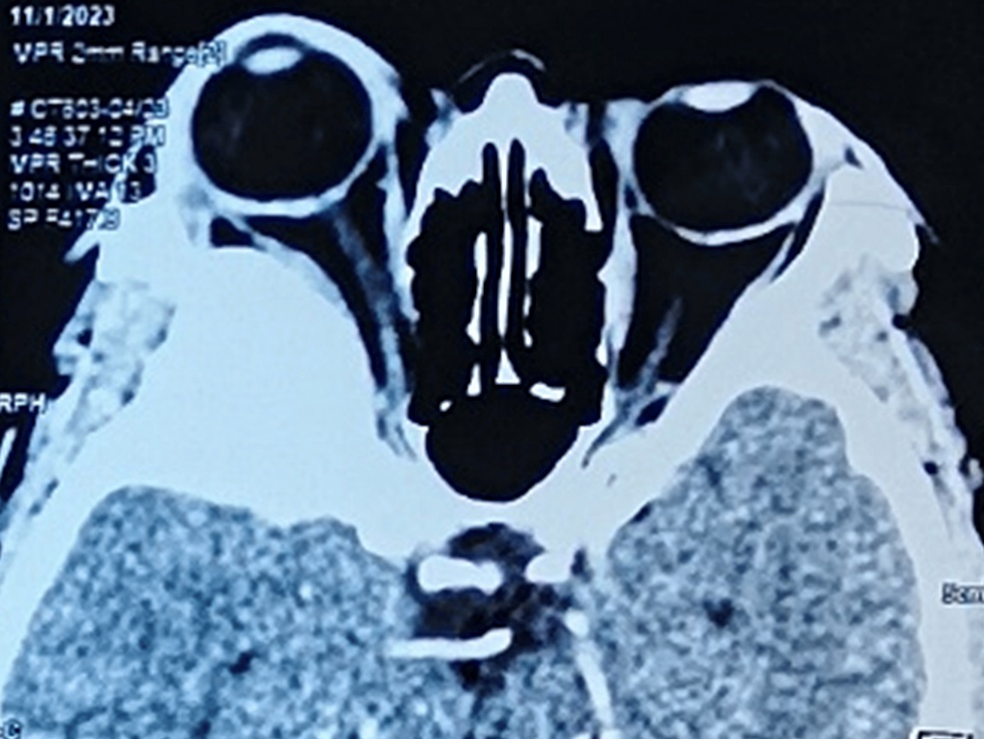
Contrast-enhanced computed tomography (CECT) shows an extra-axial lesion of the right greater wing of the sphenoid (1.1 x 2.8 x 3.0 cm) causing right-eye proptosis and compression of the bilateral intracanalicular optic nerve and optic chiasm.

Contrast-enhanced computed tomography (CECT) brain showed a post-contrast homogenous enhancement extra-axial lesion of the right greater wing of the sphenoid with compression of both the intracanalicular optic nerve and optic chiasm (Figure 4). There was another well-defined extra-axial broad-based lesion seen at the floor of the left anterior cranial fossa measuring 2.4 x 2.6 x 2.1 cm causing a mass effect to the left anterior frontal lobe in the CECT brain.

**Figure 5:**
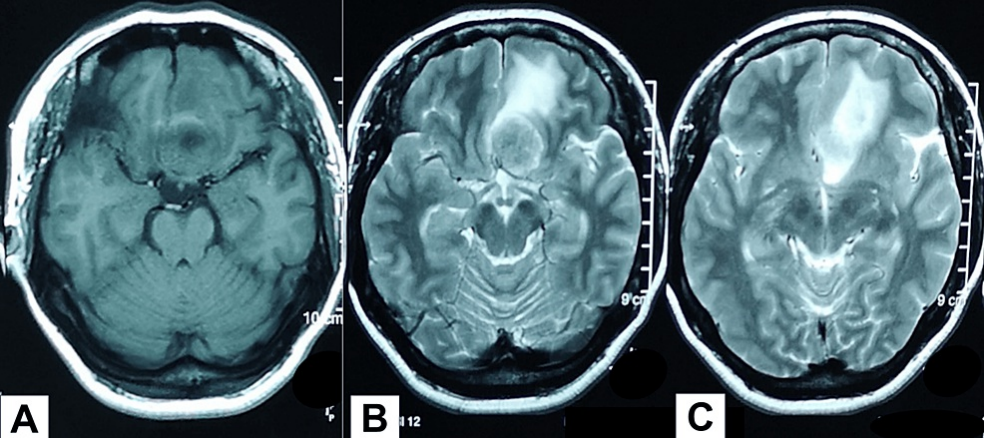
MRI shows a T1-isointense suprasellar lesion measuring 2.6 x 2.6 x 1.9 cm (A). The same lesion appeared hyperintense in T2 (B), with extension to the left anterior frontal lobe (C).

Magnetic resonance imaging (MRI) of the brain showed a T1-isointense suprasellar lesion (Figure 5A) that appeared hyperintense in T2 (Figure 5B) with compression of both the intracanalicular optic nerve (more over left) and optic chiasm with extension to the left anterior frontal lobe (Figure 5C).

**Figure 6:**
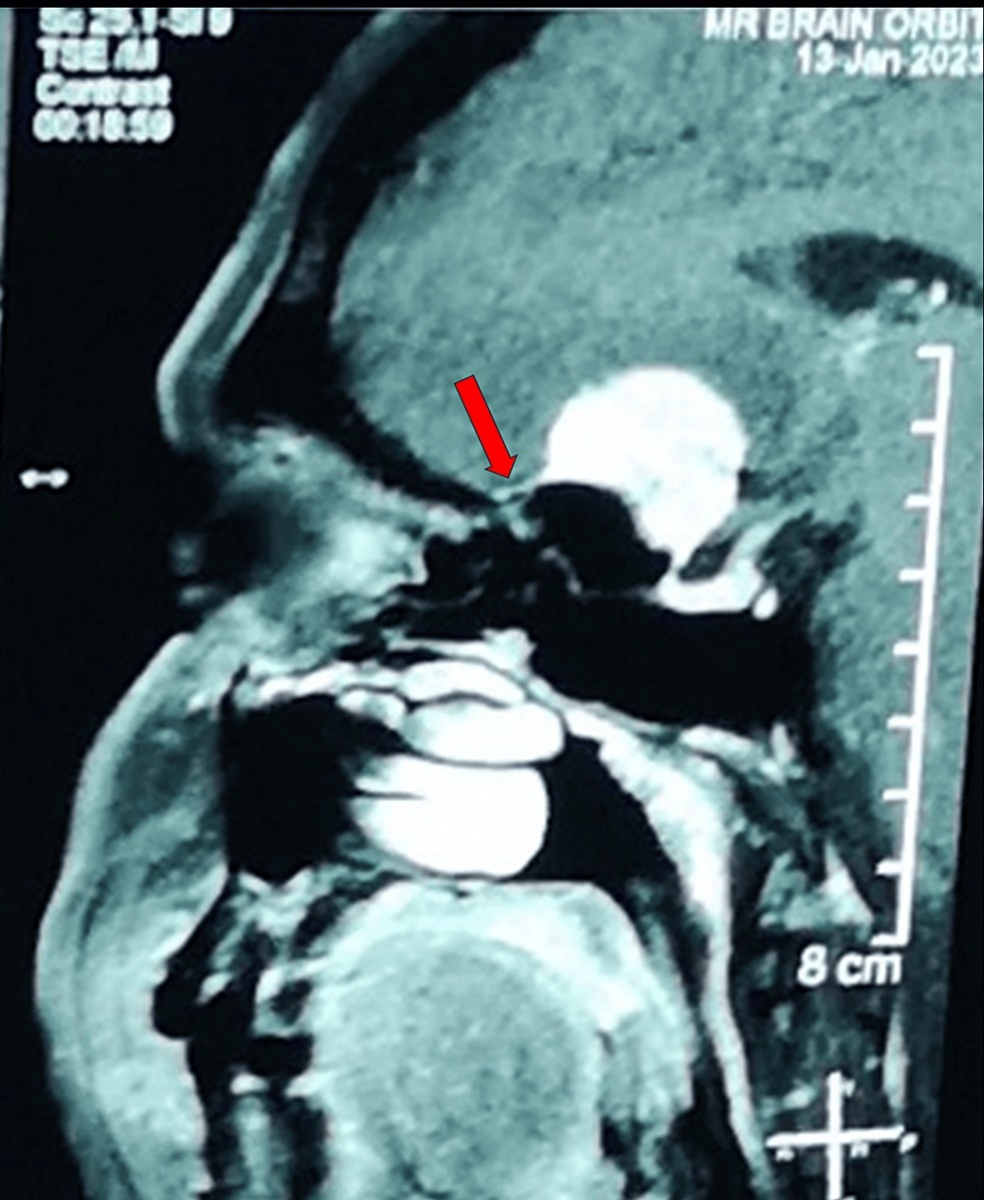
Post-gadolinium MRI T1-weighted image showed hyperintensity enhancement of the suprasellar mass with a dural tail sign (red arrow).

The suprasellar lesion also exhibited hyperintensity enhancement with a dural tail sign in the post-gadolinium MRI brain T1-weighted sequence (Figure 6).

**Figure 7:**
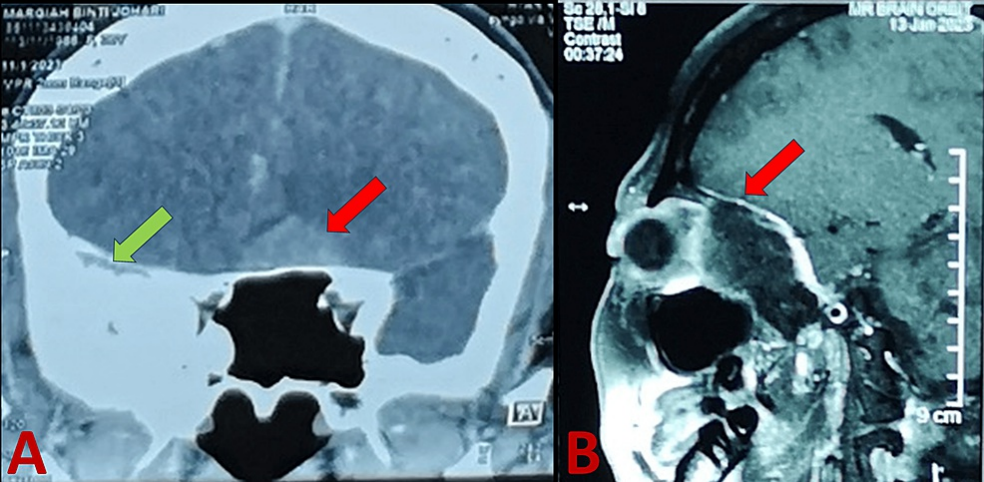
Coronal view of MRI brain T2 showed a hyperintense right greater wing of the sphenoid exta-axial lesion as shown in the green arrow and mild hyperintense sellar mass lesion as shown in the red arrow (A). MRI T1 sagittal view showed a right greater wing of the sphenoid extra-axial lesion with a dural tail sign (red arrow) extended from the right anterior part of the cavernous sinus to the right superior orbital fissure (B).

The right greater wing of the sphenoid extra-axial lesion with a dural tail extended from the right anterior part of the cavernous sinus to the right superior orbital fissure causing a mass effect to the right lateral rectus and right-eye proptosis in the MRI brain T1 (Figure 7). These findings likely represent meningioma.

She was diagnosed with bilateral compressive optic neuropathy secondary to meningioma and was referred to the neurosurgical team of Hospital Sungai Buloh in Sungai Buloh, Malaysia, for further intervention of the extensive meningioma.

## Discussion

Meningiomas are common benign intracranial tumors originating from the meningoepithelial cells of the arachnoid membrane and emerging within the subdural space [3]. Clinically, the signs and symptoms vary depending on the location involved. Typical neuro-ophthalmic symptoms associated with brain tumors encompass blurred vision, dyschromatopsia, diplopia, or oscillopsia. Common signs include visual acuity or visual field loss, RAPD, ophthalmoplegia, nystagmus, optic disc edema, ptosis, or optic atrophy [5,6]. Proptosis and vision loss are usually due to the compression of the optic nerve and extension into the posterior orbit via the optic nerve sheath or ovoid component within the orbit [7].

Approximately 15% to 20% of brain tumors (meningiomas consist of 10%) are situated in the sellar region, potentially affecting the optic nerves or optic chiasm/tract [8]. Although bitemporal hemianopia is the classic visual field defect associated with chiasmal involvement, sellar and parasellar masses can also lead to optic neuropathy (when compressing one or both optic canals), junctional scotoma (or junctional scotoma of Traquair) at the optic nerve-chiasm junction, and homonymous hemianopia in one or both optic tracts [9]. In our case, the patient was having left-eye painless vision loss since eight years ago due to compression of the left intracanalicular optic nerve by the suprasellar meningioma. Subsequently, as the suprasellar mass was growing in size and compressing, the fellow right intracanalicular optic nerve and optic chiasm posteriorly caused asymmetrical bilateral optic neuropathy (right superonasal visual field defect and left-eye severe vision loss). The right-eye proptosis was caused by another greater wing of the sphenoid extra-axial lesion.

Brain imaging is warranted when there are any ophthalmic signs of proptosis, ophthalmoplegia, and vision loss indicating any intracranial pathology. Meningiomas usually manifest as hyperdense or isodense dural-based lesions in non-contrast CT brain. In contrast-enhanced CT brain, the majority of meningiomas present as dural-based lesions with homogeneous dense enhancement, with or without accompanying brain edema [10,11]. MRI brain is the gold-standard radiological investigation for aiding in the diagnosis of meningiomas [11]. These tumors generally appear as hypointense lesions on T1-weighted and hyperintense on T2-weighted MRI brain imaging [7,10]. However, some of them may remain isointense on both T1- and T2-weighted non-contrast MRI imaging [10].

The five-year survival rate for benign meningioma accounts for 86.7%, whereas malignant meningioma has a rather poor prognosis, which is 64.6% [2]. The main treatment of meningioma is surgical removal of the lesion. The surgical excision technique of the skull of base meningioma may require sophisticated surgical instruments, such as the extended endoscopic endonasal approach due to its higher risk relationship to the adjacent bone anatomy (e.g., sellar turcica, olfactory sulcus, and sphenoid wing), cranial nerves, and blood vessels [12]. Radiation therapy is applied for tumor shrinkage in combination with surgical excision or as adjuvant therapy in postoperative recurrent meningioma [13]. Systemic treatments, such as chemotherapy and immunotherapy, are considered when surgery and radiotherapy are no longer viable options, especially in malignant or recurrent cases [13].

## Conclusions

Painless vision loss in the absence of raised intracranial pressure symptoms may be an early sign of intracranial tumors involving suprasellar mass. As the meningioma enlarges in size, it induces the late stage of ophthalmic manifestations, such as unilateral proptosis, ophthalmoplegia, and bilateral visual field loss. Early referral to an ophthalmologist and brain imaging are essential for the early detection of intracranial pathology, aiming to prevent any further vision loss resulting from compressive optic neuropathy. The gold standard for the radiological diagnosis of meningioma is MRI brain. When confronted with unilateral proptosis with bilateral vision loss, its possible differential diagnosis needs to be investigated during the planning phase for brain imaging.
